# Possible effects of cannabidiol (CBD) administration on the vocal activity of healthy domestic dogs upon their temporary separation from caregivers

**DOI:** 10.1016/j.heliyon.2024.e25548

**Published:** 2024-01-30

**Authors:** Nobuo Masataka

**Affiliations:** Center for Research of Developmental Disorders, Kyoto, Japan

**Keywords:** Vocalization, dog, Cannabidiol, CBD, Separation

## Abstract

Recent human clinical studies indicate that cannabidiol (CBD), the primary non-addictive component of cannabis, possesses considerable therapeutic potentials. The purpose of the present study was to explore the possibility of effects of CBD administration on behaviors of healthy domestic dogs. It is well-known that when temporarily separated from their caregivers, they may show an increase in vocalization. Here the effects of CBD administration upon such vocal activity were assessed. Ten 4- to 7-year-old healthy dogs experienced temporary separation from their caregivers twice, once following the administration of CBD at 2.0 mg/kg/day over a 2-week-period, and once following the administration of the same amount of olive oil as placebo over the same length of period. When the behavioral assessment was conducted by computing the total duration of any vocal activity exhibited by the dogs before the separation from their caregivers and after the separation, it was found that all of the 10 participant dogs vocalized more often when being left alone (after the separation) than when being with their caregivers (before the separation), whether they had received the administration of CBD or placebo. Following the CBD administration, however, the degree of such increase was significantly less robust than that following the placebo administration (p < 0.01). As one of the possible explanations for the results. the author hypothesizes anxiolytic effects of CBD in healthy domestic dogs, as has been reported in humans.

## 1 Introduction

Cannabidiol is the primary non-euphoric and non-addictive compound of cannabis, otherwise known as CBD. In humans, recently, it has been shown to possess considerable therapeutic potential for treating a wide range of neuropsychiatric disorders such as chronic pain [1], nausea [2], epilepsy [3]. psychosis [4], and anxiety [5,6]. A review [7] concluded that analgesic, and sedative or anxiolytic effects are exerted by CBD through its interaction with the serotonin receptor.

Besides humans, both dogs and cats are provided with such endocannabinoid system with which CBD interacts almost in the same manner researchers think it does in humans [8]. Consequently, it has recently been claimed to possess several purported health benefits in those animals, and the use of CBD-rich hemp products is becoming popular among pet owners. Typically, it is frequently claimed that CBD can be an effective and natural solution to minimize undesirable behaviors exhibited by dogs without harmful side effects, but with extremely restricted scientifically convincing evidence.

Such behaviors, being often referred to as “canine behavior problems” [[9], [10], [11]], include scratching, urine marking and spraying outside of the litter box (inappropriate urination), excessive vocalizations, etc. The present study was conceived, as a preliminary attempt, to investigate whether CBD administration could affect the occurrence of excessive vocalizations in healthy dogs, or not.

So far, it has been reported that short term oral dosing of CBD up to 20 mg/kg daily [12] does not cause any significant adverse effects in healthy dogs. A 6-month safety study found 4 mg/kg oral daily dosing [13] was well-tolerated suggesting CBD is safe for healthy dogs when fed appropriately.

In fact, a recent clinical study by repeated treatment of CBD with 20 shelter dogs with so-called behavior problems reports that their overall symptoms were improved following the treatment [14]. In the trials, the treatment lasted 2 weeks for most of the dogs, but was extended up to 6.5 weeks for the others. The findings were confirmed by the more recent anecdotal evidence [15] for the reduced aggression towards humans as a consequence of CBD administration. Another study [16] presents experimental evidence indicating that a single dose of CBD alleviates stress experienced by dogs during car travel. Importantly, however, none of these studies includes placebo control.

The current study was conceived as a blinded placebo-controlled design. Ten 4- to 7-year-old healthy dogs experienced temporary separation from their caregivers twice, once following the administration of CBD over a 2-week-period, and once following the administration of the same amount of olive oil as placebo over the same period.

As a behavioral measure to assess the effects of the administrations, the vocalizations produced by the participants were quantitatively compared between the CBD administration and the placebo administration The author hypothesized that the vocal activity exhibited by the dogs somehow differed quantitatively between the two administration conditions, it could be regarded as suggestive evidence for that CBD really affected the dogs behaviorally.

## 2 Material and methods

### 2.1 Participants

Since the current study was conceived as a preliminary attempt, the sample size was small. Ten healthy pet dogs and their caregivers were recruited through personal contact, and advertisement on a local newspaper that was published in Nago, Okinawa Prefecture, Japan where this research was conducted. The dogs were all males and had been neutered prior to study, ranging in age from 4 to 7 years. They included toy poodle (n = 2), shiba (n = 5), and miniature dachshund (n = 3). Throughout the study, all of the ten dogs were healthy and were with no medication. The author chose male dogs as the participants because excessive vocalizations were observed more often in them than in female dogs [17,18].

### 2.2 Design and administration

During the study, the participants received both a 2-week-administration of CBD and a 2-week-administration of matching placebo. Both were conducted, being separated from one another with a 30-day-interval. On the day when each administration was completed, vocal recording was undertaken with each dog, with its caregiver and without its caregiver, in order to assess the effects of the administration. The procedure of such administration was entirely blinded.

At the commencement of the present research, 5 of the 10 dogs were assigned to receive the CBD administration prior to the placebo administration while the placebo was to be administered to the remaining 5 dogs prior to the CBD administration. For the CBD administration, 2.0 mg/kg CBD was daily administered in a single dose in the morning over a 2-week period. For the placebo administration, the dogs were to daily receive olive oil as a matching placebo over the other 2-week period.

On a day when the CBD or the placebo was administered, a veterinary technician employed for this study visited the dogs at the house of their caregivers with CBD oil or placebo before daily feeding, confirmed that the dog was healthy, and administered the necessary amount of the prepared oil to the participant, using a syringe. The technician did not know about the purpose of the present research.

The CBD used was Elixinol-Entry Hemp Oil (the product of Elixinol, US) that was produced from the stalk of hemp plants. A 10-ml-bottle of the product that was for sale by the company contained 500 mg of CBD (50.0 mg/ml), but no delta-9-tetrahydrocannabinol (THC). The placebo contained olive oil. For each dog, roughly 10 ml of the CBD oil or the same amount of the placebo was re-bottled in a container that was different from that in which it had been originally bottled, and that was identical in size, color, smell, and as well as taste. The technician who visited the home of the guardian did not know the content of the container.

### 2.3 Vocal recording session and analyses

Vocal recording session with each dog was undertaken in the testing area of a house rented for this study where its caregiver visited with the dog. The testing area was a sparsely furnished room measuring 3.5 m by 4.5 m. The room had two doors without any window. Only one of the two doors was used for entrance and exit. One chair was located along a wall approximately 2 m from the door. A tripod and video camera with a microphone were located in the corner opposite the door and chair. Three toys (a tennis ball, a squeaky toy, and a rope toy) were spread out on the floor around the chair. This room and setup was novel to the dog and caregiver prior to this study.

Upon arrival at the house, the dog and caregiver met the experimenter at the entrance hall. Subsequently the experimenter led the dog and caregiver into the testing area, indicated for the caregiver to remove the dog's leash and take a seat in the chair, and instructed to “play with your dog there as usual”. The video in the room was kept on.

Thereafter, the experimenter exited the room and closed the door, leaving the dog and caregiver in the testing area for a following 10-min period.

When 10 min elapsed. The experimenter opened the door, and this time indicated to the caregiver to stand up, say “goodbye” to the dog, exit the room. The caregiver was forced to follow the experimenter out of the room, leaving the dog alone. The vocal recording of the dog lasted another 10 min, thereafter, and the entire recording ended.

For the assessment of the effects of the administration of either the CBD or the placebo upon the dog's activity, any vocalization produced by the dog that had been recorded over a 10-min-period before the separation from its caregiver and over a 10-min-period after the separation, were all submitted to soundspectrographic analyses [19], and the total duration of such activity was computed with the accuracy of 0.01 s.

## 3 Results

Table 1 presents the total duration of vocal activity exhibited by the 10 dogs when they received the administration with CBD and when they received the administration with placebo. A comparison of the vocal activity before the administration revealed no significant difference whether the dogs received the administration with CBD or placebo subsequently (t = 0.56, df = 9, p = 0.588, η_p_^2^ = 0.178). After the administration, however, the total duration of the activity when received the administration with placebo significantly exceeded that when received the administration with placebo (t = 4.95, df = 9, p = 0.00079, η_p_^2^ = 1.566).

**Table 1:** Total duration (in sec) of vocal activity exhibited by the 10 dogs when they received the administration with cannabidiol (CBD) and when they received the administration with placebo (Placebo). They were observed before and after the separation from their caregivers.

Administration				
	CBD		Placebo	
Dog	Before Separation	After Separation	Before Separation	After Separation
1	84.96	103.77	76.54	175.36
2	140.35	194.84	162.16	233.57
3	34.03	50.82	32.99	117.69
4	153.87	206.42	153.96	253.47
5	40.06	54.13	36.24	78.09
6	57.11	70.04	62.04	121.54
7	26.04	53.88	19.67	94.85
8	52.74	79.48	61.84	113.42
9	46.01	88.39	42.67	100.33
10	35.44	76.23	38.53	69.03
Mean	67.06	98.12	68.63	135.74

All of the ten dogs exhibited the vocal activity more often when being left alone than when being with their caregivers. Following the CBD administration, however, the degree of such increase was significantly smaller than that following the placebo administration (t = 4.64, df = 9, p = 0.00122, η_p_^2^ = 1.147).

## 4 Discussion

In the present study, all of the 10 participant dogs vocalized more often when being left alone than when being with their caregivers. While they were more likely to vocalize when alone, whether received CBD or placebo administration, such increase of their vocal activity was less excessive after the CBD administration as compared with the placebo administration. As already noted, the current study was undertaken as a preliminary attempt. Nevertheless, such difference was statistically significantly significant. The author considers these findings as a first experimental demonstration of possible effects of CBD administration on any behavior of domestic dogs though the population under study is still limited (10 dogs).

As a preliminary attempt, acoustic classification of the recorded dog vocalizations was not conducted here, but the effects of CBD were examined with respect to the overall vocal activity exhibited by the participants. That makes it difficult to undertake more detailed analysis on how the vocal activity could be modulated by its administration. Nevertheless, the fact should be noticeable that pet dogs often become agitated by their caregivers’ departure or absence with separation anxiety. Usually, right after a caregiver leaves a dog (often within a few minutes), the dog will begin intensely vocalizing [17,18]. As long as such frustrated response is short-term one, it is healthy [20]. However, when the response is prolonged (e.g., becoming panic every time a caregiver leaves), chronic stress may manifest itself as pathologic symptoms [10].

In humans, a pioneering study investigated effects of CBD on people with social anxiety disorder (SAD) who were public speaking, and found that those people who were treated with CBD had reduced anxiety and improved comfort when they were speaking [21]. More recently, the author recruited 37 Japanese teenagers with SAD who were randomly allocated to receive treatment with either CBD or a placebo for 4-week-treatment [22]. When anxiety was measured before and after treatment, using the Fear of Negative Evaluation Questionnaire and the Liebowitz Social Anxiety Scale, the teenagers who received the treatment with CBD had significantly lower scores in both of the anxiety scales when compared with the teenagers who received the placebo treatment. The present results in dogs are consistent with the findings reported in these two studies.

Importantly, however, any information is not provided about the emotional state of the dogs in the current study when they were alone, but they were apparently healthy dogs without behavioural problems. Therefore, we are unable to infer any clinical benefit such as anxiolytic effects from CBD. One of the most common behavioural problems in dogs are separation-related problems and the most common symptoms include excessive vocalizatons. Therefore, ‘separation anxiety’, often used as a synonym for all separation problems, refers to a dog's behaviour including sign of anxiety or fear when separated from the caregiver. Nevertheless it appears necessary to keep in mind that all separation-related problems are not caused by anxiety or fear. As destruction behaviour can be motivated by overactivity, play, or boredom [23], vocal activity could also be a reaction to external stimuli, or it can even be socially facilitated [24].

Taken together, such reasoning would lead us to assume that sedative effects of CBD could be exerted in healthy domestic dogs, as has been reported in humans [25]. This should apparently be the issue to be investigated in more detail in the near future.

## Statement of ethics

This study protocol was reviewed and approved by the Institutional Ethics Committee of Center for the Study of Developmental Disorders, approval number [ [21]-01]. The dog-caregiver dyads participated on a voluntary basis. Informed consent was obtained from the caregivers.

## Data availability statement

Data are included in the article. Additional data will be made available on request.

## CRediT authorship contribution statement

**Nobuo Masataka:** Writing – review & editing, Writing – original draft, Investigation, Data curation, Conceptualization.

## Declaration of competing interest

The author has no conflict of interests to declare.
